# Lost in the shuffle: A taxonomy for the accumulation of unwanted elements in steel recycling

**DOI:** 10.1177/0734242X251350541

**Published:** 2025-07-28

**Authors:** Beatriz Pérez Horno, Andreas Feldmann, Peter Samuelsson

**Affiliations:** 1 The Department of Industrial Economics and Management, KTH Royal Institute of Technology, Stockholm, Sweden; 2 The Department of Materials Science and Engineering, KTH Royal Institute of Technology, Stockholm, Sweden

**Keywords:** Circular economy, resource management, impurity, recycling, proactive approach

## Abstract

Aiming to reach circularity and resource efficiency, the metal industry pushes towards recycling secondary products instead of producing from primary material. This increases the use of scrap, which can bring about several benefits but could also come at the expense of the materials’ quality and the potential loss of valuable resources. The above is mainly a result of the likely accumulation of unwanted elements throughout the recycling process, which may dissolve and become difficult to extract from the metal’s melt, turning into what is known as tramp elements. This study focuses on the opportunity to limit the accumulation of unwanted elements before they end up in the molten solution. Taking an exploratory approach with the use of observation and expert interviews, this study examined where and how unwanted elements enter the recycling system. Eight element types were identified and categorised after the intentionality of their addition and their desirability in the end product. Thereafter, this article proposes a taxonomy based on the way in which these are present inside the furnace before melting, suggesting that the manner elucidates the entry points where impurities are introduced in the recycling stream. By introducing a taxonomy, this study aims to pave the way for developing strategies and research on how to minimise or prevent the presence of these elements in recycled metals, thereby increasing the quality of the recycling process.

## Introduction

The depletion of available natural resources and the accumulation of waste on the land’s surface instigate the transition to circular industrial systems and resource efficiency, where materials and other resources circulate at their highest value within the planetary boundaries (Gaustad et al., 2018; Tan et al., 2021). Recycling poses an opportunity to address the above, reducing material and production costs while retrieving valuable elements (Broadbent, 2016). Metals are often presented as an example for a continuously recyclable resource (Bartl, 2014; Grosso et al., 2017). Yet, in practice, this is not entirely correct. The presence of metals in various complex alloyed forms, the imprecise dismantling and sorting mechanisms and the incomplete awareness on scrap’s composition can lead to the mix of disparate metal grades, alloying elements and sometimes non-metallic materials inside the furnace (Awasthi et al., 2017; Daigo et al., 2017; Panasiuk et al., 2022). Scrap then consists of separate pieces, each with a unique chemical composition and varying contamination levels (Björkman and Samuelsson, 2014; Harvey, 2021). Some may even be pure materials, but of a sort that is unwanted in a particular product, such as, for example, copper wire in steel scrap (Daehn et al., 2019). Once exposed to high temperatures, these elements become evenly distributed in the melt, turning into what is known as tramp elements, contaminants or impurities in the recycled material (Cavaliere, 2019; Ciacci et al., 2022). This implies a double loss for the industry. Firstly, it degrades the quality, purity and properties of the material that is to be recycled (Miranda et al., 2019; Nakajima et al., 2018; Tan et al., 2021). And secondly, it induces the loss of valuable (and often scarce) resources present within, which can no longer be recovered (Diener and Tillman, 2015; Graedel et al., 2022). Therefore, even materials with the theoretical potential for infinite recyclability are limited by the inefficiencies of current recycling processes (Grosso et al., 2017). These losses have significant environmental and economic repercussions, contributing to resource depletion, environmental degradation and pollution. Economically, valuable resources remain underutilised while additional costs are incurred in removing impurities. As valuable resources are lost or degraded during recycling, industries may need to rely on more expensive virgin material. Moreover, additional processes and purification steps might further raise costs. Degraded materials can reduce the value of the recycled output resulting in lower returns and missed opportunities for revenue generation. From an environmental perspective, depending on primary materials increases energy consumption and pollution. The additional processing might also lead to higher energy consumption and pollution.

An element present in the metallic melt can be an impurity or an alloying element. An impurity is detrimental to the metal, while an alloying element is added to achieve specific properties in the final product. The objective is to reduce impurities to the greatest extent possible. Thus, what strategies can be employed to effectively reduce the quantity of impurities in the recycled material? In the case of steel, there are three primary approaches. Firstly, impurities can be removed through standard metallurgical practices, such as oxidation to slag or off-gas dust. Secondly, these can vaporise and oxidise during melting. For instance, zinc does not dissolve in the metallic melt but oxidises and becomes dust. Thirdly, for those elements that cannot be removed through the previous processes, dilution with the addition of relatively pure steel scrap can be a solution. The first and third approaches can also be applied to address an unwanted excess of an allowing element. Although these strategies are useful, this article aims to explore and question the possibility of reducing or limiting impurities before the melting stage, adopting an operational and process-oriented perspective.

The notion of material quality degradation and/or contamination is not new. Research on so-called tramp elements in metal scrap has made significant progress in the past decades. Although the definition of tramp elements remains inconsistent, the research on tramp elements is commonly characterised by (1) their presence within the chemical structure of the recovered material (Ciacci et al., 2022), (2) their difficult and costly extraction (Björkman and Samuelsson, 2014; Cavaliere, 2019), (3) the harmful effect on the material in which they reside (Bartl, 2014; Ohno et al., 2014), (4) being unwanted (Grosso et al., 2017; Panasiuk et al., 2022) and (5) the challenges when they are present at intolerable percentages, exceeding the maximum allowed concentration (Daigo and Goto, 2015). In this article, the word tramp element refers to all *unwanted* elements that remain *inside the metallic melt* of the recycled material, as they are hard to extract in a cost-efficient way. From this approach, although different elements can be unwanted in a certain material, only those inside its chemical composition can be considered tramp elements. This distinction between tramp elements and unwanted elements is essential to the article’s argument. Previous literature has extensively examined the chemical composition and the presence of certain tramp elements in recycled products and by-products (Ciacci et al., 2022; Daigo and Goto, 2015; Ohno et al., 2014; Panasiuk et al., 2022). However, less attention has been taken to examine how these resources end up in the scrap stream. Since recovering these resources after melting is costly and induces resource losses, limiting the mix of alloys prior to melting could be a valuable solution worth further examination (Material Economics, 2016). Although material losses and contamination seem inevitable in practice (Bartl, 2014), here we assert these may be minimised when identifying and acting upon the anthropogenic mixing of elements throughout the whole recycling process.

Taking a process approach, this work:

1. Identifies how elements are present before melting
2. Identifies where in the process different elements enter and mix
3. Proposes a taxonomy framework based on the above

Looking at the accumulation of unwanted elements throughout the recycling process, this article proposes a taxonomy framework that distinguishes the diverse types of elements present in the pre-melt stockpile at the furnace, suggesting that the way in which they are present elucidates the entry points where impurities are introduced in the recycling stream. This pursues to help develop strategies for contamination prevention, quality control and resource management in metal recycling operations.

## Methods

This article sets out to detect the sources of entry of (unwanted) elements and the manner in which these are present inside the furnace before melting. This study builds upon and extends previous research conducted by the co-authors (Berlin et al., 2022; Compañero et al., 2023, 2024). Since these studies investigated the same system, both in general terms and in more detailed scrap handling procedures, it allowed for more focused data collection for this article. This work combined direct observations of scrap yards and mills, and interviews with industrial and academic experts. The retrieved information was subject to a thematic analysis to formulate a (new) taxonomy framework (Braun and Clarke, 2006).

The dataset consists of 3 on-site visits to steel mills and recycling centres and 14 expert interviews. The use of expert interviews was critical for obtaining detailed, real-world insights into a complex issue. However, the topic is of sensitive nature to the organisations involved. To address confidentiality concerns and the potential risks associated with discussing operational details, participants were assured anonymity, collecting valuable data through note taking. While this inevitably affects the breadth and generalisability of the findings, the study provides a foundation for understanding and organising the phenomenon, highlighting areas for future exploration. The interview guide can be found in the Supplemental Material and Appendix. The interviews provided detailed accounts of the accumulation of unwanted elements throughout the different steps in the recycling process, with recurring themes emerging early in the data collection process. Although the sample size is modest, the consistency of responses and experiences across participants suggests that the data are conceptually sufficient to address the research objectives. Similar qualitative studies in industrial settings (e.g. Guest et al., 2006) demonstrated that a sample size of around 12–14 interviews can be sufficient for data saturation. However, we acknowledge the limitation of sample size and suggest future research with broader datasets.

This process took place between October 2022 and March 2023. During this time, the main author visited three scrapyards: two steel mills and one scrap dealer. These visits enabled the identification of certain patterns and challenges in the dismantling, shredding, sorting and recycling process, and in material accumulation and allocation overall. Moreover, they provided an overview of the scrap yard distribution, functioning and grading system, as well as insights into the decision-making process at each stage. Besides, they provided practical examples that aided the categorisation and elaboration of groups. To expand the knowledge on scrap and uncover those facts difficult to perceive through observations, the previous process was supported with expert interviews. The interview process started with dialogues (group interviews) with scrapyard managers, scrap purchasers, and furnace analysts and managers from two different international mills. These enabled a deeper understanding of the input and output materials while providing further insights into the recycling process. For a better picture and to expand the perspective in which scrap is looked at, the study also incorporated the point of view of the scrap dealers. The addition of these other site visit and interviews to the sample filled in some missing information on scrap and the entry of elements. Thus, metallurgists, experts in steel and researchers in material recycling from various disciplines and backgrounds, were approached during the data collection. Altogether, a total of 14 semi-structured interviews (with a duration of 30–60 minutes) were performed with professionals from diverse backgrounds and expertise, each of which was shaped according to the interviewee’s profile. Thereafter, the study benefited from the perspectives of a cross-disciplinary sample of experts that reduced bias, increased validity and enabled them to obtain retrospective and real-time accounts from those experiencing the phenomenon of tramp element accumulation (Alvesson and Sköldberg, 2009; Gioia et al., 2013). Due to participants’ preferences and confidentiality, the interviews were not audio-recorded. Instead, detailed notes were taken during and immediately after each interview to capture the participants’ responses and observations. Even though we recognise the limitations from such an approach, the method provided sufficient valuable insights into the phenomenon under study. Immediately after the interviews, the individual notes were reviewed, shared and combined with those of the different researchers participating in the process. To enhance validity, summaries of the compiled scripts were shared with the participants for confirmation and clarification. These summaries are available upon request. Moreover, the compiled data were verified against the existing literature.

Conclusively, the data collection process followed an iterative structure of going back and forth to the different experts and literature. The triangulation of the data from observations, interviews and existing literature further strengthens the validity of the findings. The data collection took place in central and north European steel mills and sorting facilities. Thus, the findings should be interpreted with caution and might not fully capture the breadth of perspectives within the broader population. Although the comparison of results with other literature suggests that these findings could be applied to other metal recycling, it should be noted that the taxonomy provided here represents the accumulation of unwanted elements in metals during the recycling process in north and central Europe (Table 1).

**Table 1.** Summary of on-site visits and expert interviews.

Organisation type	Number of on-site visits	Interviewee’s expertise	Number of interviewees
Steel mill	2	Raw material purchasing	3
		Scrapyard manager	2
		Furnace manager/analyst	2
Scrap dealer	1	Experience with scrap sorting	3
Other		Metallurgist and expert in steel	2
		Researchers in material recycling	2

The information extracted from the observations and expert interviews were analysed using Braun and Clarke’s (2006) six-phase thematic analysis framework. Once familiar with the data, the first-order codes were generated (Braun and Clarke, 2006; Gioia et al., 2013). The initial codes were built through manual line-by-line analysis of the notes to identify patterns of meaning across the dataset. The codes were descriptive and focused on capturing repeated ideas and experiences, such as frequently finding copper wires in steel scrap or the recurrent process and/or solution of diluting scrap with virgin material to reduce the percentage of impurities rather than removing these from the molten metal. Furthermore, the codes were grouped in themes after a comprehensive and iterative process of synthesising insights from the data. This means that while codes served as a foundation, themes were created by identifying patterns of meaning and relationships that extended beyond the participants’ direct statements. Nonetheless, formulating themes that enabled answering the research objective from the data required a deeper level of abstraction. For example, from the insight ‘we mix different metal grades to meet the specifications’, the authors interpret that the mixing of metal grades is intentional despite knowing these will bring both wanted and unwanted elements to the final metallic melt. Thus, the theme framing process focused on determining the inflow and outflow of (unwanted) material in the system. This process uncovered two key aspects of these inflow and outflow, which is named in this study as ‘intentionality’ and ‘desirability’. If we take an example from interviewee B ‘Titanium coming from paint in white goods will end up in the slag’, we conclude that the input is in the surface treatment and the output is slag. This interviewee also said ‘30% of the copper content melted in the steel is originated from copper cables not sorted in scrap’. Here, we extract that copper is introduced unintentionally, which means that it is not a consequence of a premeditated voluntarily decision. The emergent themes were cross-validated by the research team and compared and reviewed iteratively with the existing data and literature until reaching a proper category definition and naming (Alvesson & Sköldberg, 2009; Braun and Clarke, 2006). The identified categories and determinants are synthesised and compiled in the tentative framework (Alvesson & Sköldberg, 2009).

## Results

This work examines the material accumulation and loss within the system. Regardless of the disparate backgrounds and disciplines, experts coincide in (1) the cumulative nature of wanted and unwanted materials in the scrap piles; (2) the shared unawareness of the chemical composition of scrap throughout the process and the problematic consequences of this, and (3) the behaviours and processes that lead to the mixing and entry of elements in the system.

**Figure 1.**
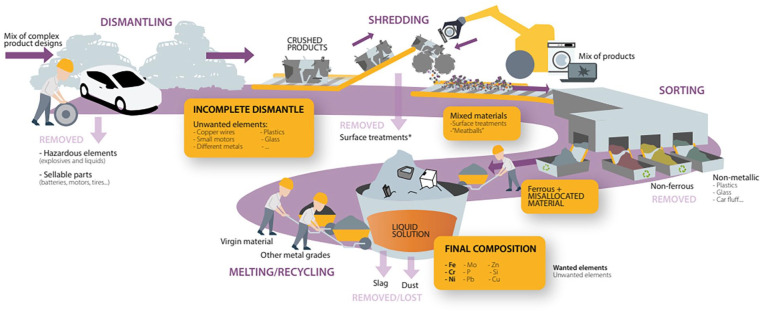
Inflows and outflows of (unwanted) material and the accumulation of these during the dismantling, shredding, sorting and melting process.

The system spans from the reception of end-of-life products at the dismantler to the melting and recycling of metal scrap at a steel mill. Although not all products experience each and every step described below, this section covers all plausible processes involved in the management of metal scrap. End-of-life products collected from both consumers and Original Equipment Manufacturers (OEMs) typically undergo a conjunct process of dismantling, shredding, sorting and recycling. Figure 1 shows a graphical synthesis of inflows and outflows of (unwanted) material and the accumulation of these during the dismantling, shredding, sorting and melting process. Each of these stages in the value chain is subject to the mix and/or loss of valuable resources, ergo contributing to the accumulation of (unwanted) elements and the consequent degradation of the material’s quality. Considering the scrap’s composition at the mills is uncertain and costly to analyse, discerning the mechanisms and steps in the value chain by which impurities are introduced requires tracing back and examining the preceding processes, systems and flows.

### Accumulation during product dismantling

Once the collected end-of-life products reach the dismantler, most parts that are either hazardous (e.g. liquids and explosives) or economically attractive and saleable (e.g. batteries and motors) are removed. As the dismantler’s profitability relies on tonnes of scrap sold, once these elements are extracted from the product, the remaining are compacted, for example, flattened together, to the furthest extent. Some removable parts are not extracted and could damage the following processes. Taking a car as an example, small motors and/or copper wires can be left in the crushed material sent to the shredder/sorting. The extent to which removable parts are detached is curtailed by the complexity in which product parts are designed and assembled, and the profitability of the dismantling process. Thus, if small motors are allocated in areas that would increase the time of separation, these are perceived as not worth removing and will in the best case only be separated after the shredding process. Thereafter, the outcome of the dismantling process is a compact mix of products and materials (plastics, glass, metal alloys, etc.) hard to separate from one another.

### Accumulation during material shredding and sorting

A combination of materials and products coming from different sources are fed together into the shredder to economically optimise the process (e.g. flattened cars with white goods). The shredder is a well-developed, automated process that reduces the material to a particle size suitable for sorting. However, feeding the machinery with unwanted material (e.g. copper, aluminium or high alloy steel) can thwart the recycling process. There is a trade-off between product quality and productivity. The more homogenous the input is, the more homogenous the output becomes. Through smaller batches with products that are known to have similar material compositions, the mixing of materials would be reduced. However, the machinery can process materials more efficiently, if it is loaded with a mix of products of complementing sizes. The pace at which the shredding is conducted has an impact on quality and increases the risk of entangled materials in what is called ‘meatballs’, hindering their separation. An observed example was a small electromotor encircled and crushed together with copper wires. Surface treatments (such as paint) can also be removed in the shredder, but this is not always achieved. Therefore, the accumulation of material at this stage is mostly a consequence of the fast operating rate and/or the incomplete dismantling of complex products that propel the problem as it advances in the value chain.

Consecutively, different sorting processes can take place. Examples are magnetic separation, density separation or the use of air, in the case of light materials. Focusing on metals, these are segregated into ferrous and non-ferrous with the use of magnetic separation. Although the system seems optimal, some non-metallic materials can indeed mix with the metallic. Following the example of the entangled products, as these flow through the sorting process, they inappropriately follow together a unique material stream, misallocating some metal alloys in the wrong scrap pile. Thus, a mix of various material types (especially metallic) arrives at the same scrap pile sent to the mills. This brings in disparate alloying elements that are neither intended nor wanted in the secondary material.

### Accumulation during material recycling

The remaining pile from previous processes blends inside the furnace. To optimise the melting process and reach product specifications, mills develop a recipe that combines different material compositions or grades, while ensuring the compound stays within the specific contamination tolerances at the lowest production costs. Current recycling processes cannot rely on scrap as the only material source. Virgin material is added to the melt to compensate for and readjust the chemical composition below the tolerated contamination levels. Diluting impurities is a standard solution for those elements that cannot be metallurgically extracted. Although this solves the symptomatic problem temporarily, these elements are not removed from the compound and therefore carry on accumulating and increasing their presence cycle after cycle. Besides, even virgin materials can be sources of small amounts of contamination, such as phosphorus. Consequently, contamination comes from all three input materials: virgin raw material, sorted scrap and the different metal grades. Although some elements come from iron ore, most derive from the last two material sources. The first includes tramp elements from previous lifecycles and unwanted elements from the incomplete dismantling and sorting processes (e.g. coatings and paintings). On the other hand, other metal grades include alloys wanted in the end product, yet they most certainly contain other alloying elements that are not always desired (e.g. molybdenum and nickel) and can harm the secondary material. Once all material melts together, most wanted and unwanted elements dilute in the metallic melt, getting trapped and accumulating inside this. Their solution is then irreversible and when not removed, these will carry on accumulating every cycle until they reach intolerable percentages and the product can no longer be used and becomes obsolete.

On that account, the major causes of unwanted material accumulation lie in (1) the complex design and assembly of components in a product, (2) the broad range of new materials and alloying combinations, (3) the financially driven processes and consequent misallocation and mixing of material and (4) the low awareness on scrap’s composition. Despite the willingness to transition towards more sustainable ways of producing steel, the industry shows concern about the use of scrap in their production due to the quality implications. To avoid this from reproducing in the future, experts have highlighted the need to conduct a more careful separation before the shredding process and a more conscious material selection and product design.

### Material accumulation also leads to resource loss

**Figure 2.**
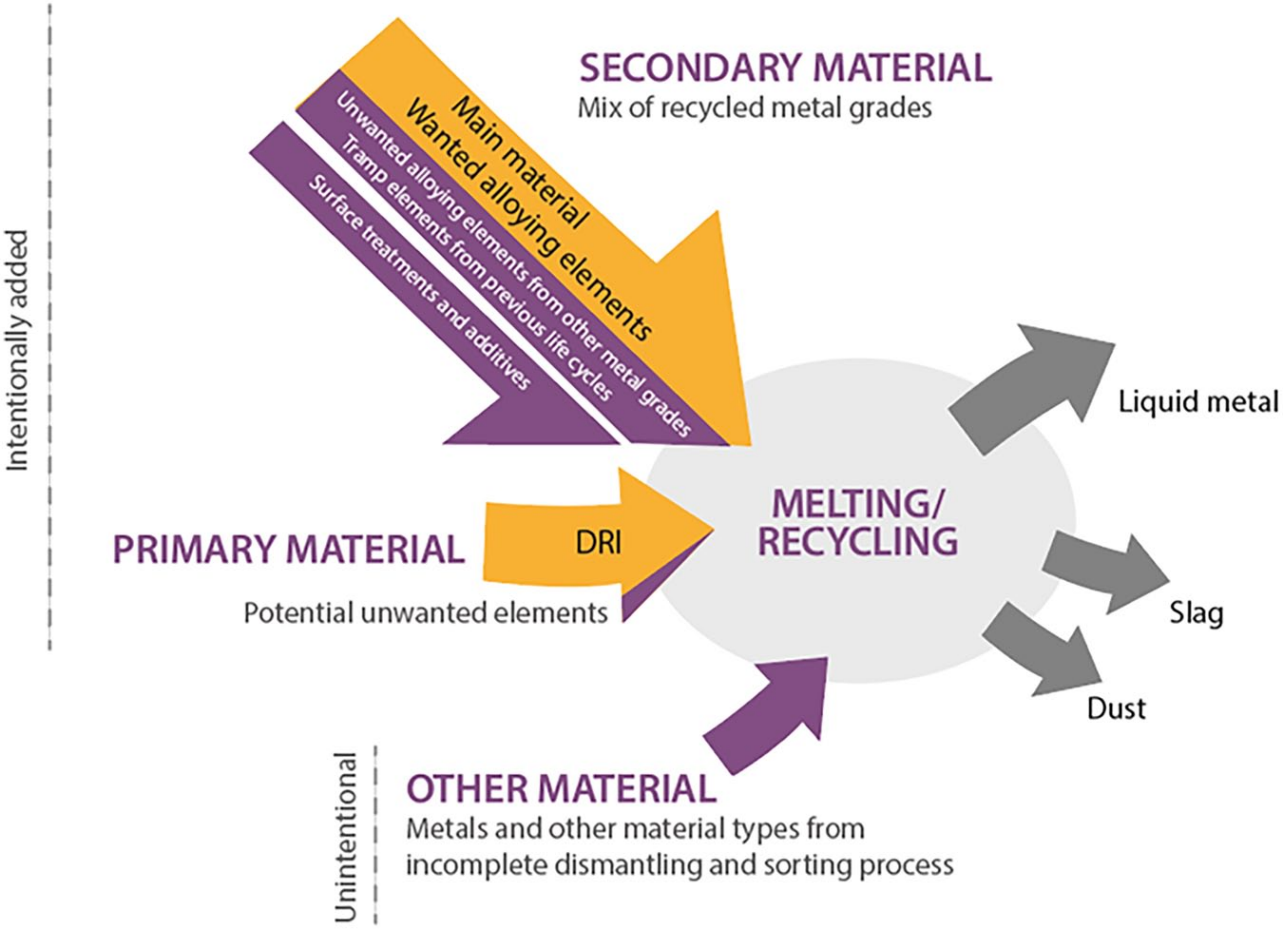
Overview of the input and output material and products at the furnace.

As seen above, the recycling process includes virgin/primary material, secondary material and other unintentional pieces from the incomplete dismantling and sorting process as input, see Figure 2 for an overview of the input and output material and products at the furnace. The primary material merely contains the main material or alloys, whereas the secondary material can also include surface treatments such as coatings and paintings and/or some additives, along with some unwanted encapsulated elements from previous life cycles, such as copper, nickel or a small fraction of molybdenum, depending on the steel grade produced. The unwanted elements that arrive at the scrap pile after the imperfect sorting mechanisms can be other metal grades or other material types. Lastly, the intentionally added metal grades contain both – wanted and unwanted elements. Table 2 shows supporting statements from interviewee’s in the characterisation of the different element types. All these elements melt together in the steel-making process, resulting in the desired metal and the unavoidable by-products: slag and dust. Thus, the array of elements gets distributed among these three outputs, bringing about the downgrading of the recycled material and the loss of valuable resources. The chemical composition of the molten metal is analysed after this process and to readjust and meet the product’s specifications virgin material are typically added. Yet, those elements trapped and present in smaller quantities are hardly extracted and remain for future life cycles. Therefore, these elements not only are seen as contaminants but also entail a resource loss. This loss is twofold. Firstly, it is a loss of the trapped elements per se. Yet, it is also a loss of the main material content in the product (iron in the case of steel). The higher the content of contaminants in the compound, the lower the amount of iron, which involves adding extra iron to meet the new product specification, turning into higher costs. The loss of resources is also experienced with the by-products from which only iron is recovered. Although slag is needed for the metallurgical process, both slag and dust are not valuable for the mills, entailing a loss of materials, especially oxides (e.g. manganese). Moreover, some other products can bring about material losses. This is the case of plastics, which will not contaminate the product but will evaporate or oxidise in the process; or surface coatings (e.g. tin and zinc), which tend to end up in dust.

**Table 2.** Characteristics of the different element types. Interview guide and list of interviewees can be found in the Annex.

Category	Characteristics	Some supporting participant statements
Surface treatments and additives	Not wanted yet these mostly oxidise or evaporate in the melting process.	Interviewee A: ‘The piece is likely to also include other elements that derive from potential coating and paint layers, and some other contaminants that were accumulated during their recycling, or in previous life cycles’.
		Interviewee L: ‘There are many painting that are polymeric and most will evaporate or oxidise’.
		Interviewee M: ‘Additives transfer mostly into the slag and not into the steel’.
Potential unwanted elements in primary material	Not wanted yet unavoidable as they are already present in the primary material.	Interviewee A: ‘However, the presence of some impurities, such as phosphorous, seems to be inevitable regardless of the input scrap’.
		Interviewee I: ‘Phosphorous coming from iron ores that include some alloying elements’.
Other metals from incomplete dismantling and sorting process	Not wanted, yet a better sorting can avoid them from entering the furnace.	Interviewee I: ‘A tramp element can be copper. However this is less present thanks to slight improvements in the sorting process’. Interviewee J: ‘what needs to be improved is the sorting and separation before feeding the material to the shredder’.
		Group interview A: ‘If ferrous and non-ferrous are attached very hand in the design, the ferrous material will win in the sorting and therefore there will be some non-ferrous material in the ferrous scrap. Welding ferrous and non-ferrous is very problematic; however this is not very common’.
Unwanted alloying elements from other metal grades	Mixing other metal grades in the furnace is a major contribution of tramp elements. This can be avoided at the furnace when deciding not to mix different metal grades.	Interviewee K: ‘Most trace elements come from previous alloying elements that are not wanted anymore’.
		Group interview A: ‘There is the possibility that other steel grades, metal grades or even other material families end up in the 304 blend. (. . .) Examples can be similar steel grades in the 300 series, such as 302 or 316, which can cause the introduction of elements like molybdenum. At the same time, other metal grades can be included, such as steel grades of series 400 or other alloys’.
Tramp elements from previous life cycles	Because of the previous, tramp elements accumulate over cycles. These cannot be removed and will continue to downgrade the quality of the material with every cycle.	Interviewee A: ‘The piece is likely to also include other elements that derive from potential coating and paint layers, and some other contaminants that were accumulated during their recycling, or in previous life cycles’.
		Interviewee K: ‘Copper wires are challenging as you can’t reduce copper from steel. Next time the secondary steel already has copper, once you recycle it 20 times, the copper content is too high’.

## Discussion

The loss and accumulation of certain elements in scrap are unavoidable consequences of the melting of heterogeneous material sources (Bartl, 2014). Although some of these elements were already inherent in the scrap’s molten stage before melting, others are introduced throughout the recycling process (Daigo and Goto, 2015). The following subsection proposes a simplistic taxonomy for the accumulated elements in the scrap before it is molten according to the intentionality of their addition and their desirability in the recycled output. This classification concludes with eight element types that summarise the different ways in which these elements can appear inside the furnace. Moreover, the way in which they are present suggest potential entry points by which these could have been introduced in the system, which in turn opens up more options to avoid the problem.

Some elements that end up inside the furnace are detrimental and not required or wanted in the demanded specification of the new product (Daigo and Goto, 2015). Therefore, preventing these from entering the melting stage is paramount. Although some material unintentionally ends up inside the furnace (e.g. entangled and misallocated material from dismantling and sorting processes), some others are intentionally added but not wanted (e.g. additional metal grades that include not only wanted alloys but also unwanted elements and surface treatments). Thus, the findings from this study highlight three key aspects:

1. The intricate nature of alloys which can be both wanted or unwanted. One example is when introducing other metal grades inside the furnace to meet the specifications. By intentionally adding this specific scrap piece that includes a desired element (e.g. molybdenum), other elements can enter the mix (e.g. some copper trapped inside). Mixing different metal grades offers opportunities to create custom alloys, reduce costs and converse resources but presents challenges in quality control and contamination. This stresses the need to analyse the composition of the grades that are introduced in the furnace to assess whether their inclusion pays off the additional elements that can harm the overall product.
2. The need to beware the creation of novel and complex alloying combinations and designs that can be counterproductive for the recoverability and recyclability of end-of-life products.
3. The detrimental nature of some elements already encapsulated in the metal from previous cycles. Some scrap might be too contaminated for certain applications from the start. An example is construction scrap, which has a higher contamination tolerance and thus can contain higher quantity of unwanted elements in the scrap. When the avoidable elements are not properly and timely removed, these accumulate in the product and end up falling into the category of ‘encapsulated material’, contributing to the downgrading and loss of material.

**Figure 3.**
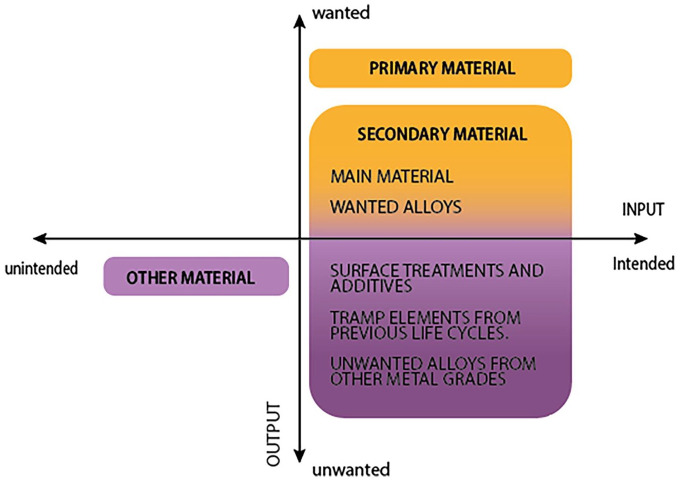
Categorisation framework for the different element types present in scrap.

Figure 3 provides a framework that classifies the different element types in eight groups distributed across the diagram based on the intentionality of their addition and their desirability in the recycled product. Thus, the framework proposes a taxonomy for all elements within, giving bigger attention to those that are potentially going to become tramp elements, based on the way in which they are present pre-melting inside the furnace (e.g. if they are coatings, alloys, misallocated metal pieces, etc.). In addition, the way in which they are present gives a hint on where in the value chain they could have entered the system. Intentionality and desirability are interlinked concepts that shape the dynamics of metal scrap recycling. Intentionality reflects the purposeful actions of recycling stakeholders, whereas desirability reflects the willingness to have these in the final metallic melt. Although the desirability of the element inside a product suggests whether this becomes a tramp element or not, the intentionality suggests if they are a consequence of current practices and decision-making strategies and dynamics. Looking at the framework in Figure 3, all element types that fall into quadrants 3 and 4 are unwanted and therefore considered as potential tramp elements that require attention in the development of contaminant prevention strategies.

## Conclusion

If the use of scrap for developing new products continues to increase, preserving the material’s quality and maintaining reliable information about its chemical composition before processing in the furnace becomes imperative. Given that the analysis and recovery of resources post-melting is potentially costly and inefficient, the quality of scrap must be enhanced at earlier stages in the recycling process. For this reason, this study proactively investigated where and how in the value chain the accumulation of unwanted materials takes place.

To begin, this study expands upon the existing body of knowledge concerning the form and entry points of tramp elements, recognising the importance to identify these prior to their dilution. Unwanted elements could manifest in all three primary input categories: primary material, secondary material and other materials. While the latter group is always unintentional and unwanted, both primary and secondary materials contain a broader spectrum of element types. The mix of secondary material grades consists of the main material, wanted alloys, tramp elements from previous life cycles, unwanted alloys from other metal grades and surface treatments and additives.

Secondly, this study corroborates that the accumulation of certain unwanted materials happens across all stages in the recycling process. The article maps current practices and points out the origins and entry points in the value chain where (unwanted) material flows in and out, revealing how these practices influence tramp element accumulation. This study displayed how productivity concerns are prioritised over reducing the accumulation of tramp elements, once the quality of the material is within the specified tolerances. Although this last statement has already been part of the discourse, recognising the specific decisions and anthropogenic actions that lead to the mixing of material may enable to take proactive action in minimising material contamination. In order to address strategies for optimising recycling processes and minimise material losses and quality degradation, this article suggests that transitioning from a cost-saving approach to one that prioritises preserving the intrinsic value of materials. Finer sorting techniques, avoiding the mixing of scrap grades that could introduce unwanted elements and implementing stringent quality control measures – can significantly reduce the quantity of impurities in recycled materials.

Finally, this article proposes a categorisation framework that generalises the findings from the study. This tentative taxonomy portrays the way in which the different elements appear pre-melting inside the furnace, discerning those elements that are ought to become tramp elements. Although the framework is developed only based on recycling of steel, the underlying principles and method for application can be used to study other materials that struggle with the accumulation of impurities, for example, aluminium and plastics.

Overall, this study narrows the knowledge gap on the presence and introduction of unwanted elements in the system. By introducing a taxonomy for the accumulation of unwanted elements, this study aims to pave the way for developing strategies and research on how to minimise or prevent these elements’ presence in recycled metals, thereby increasing the quality of the recycling process. This endeavour aligns with the broader goals of contamination prevention, quality control and resource management in metal recycling operations. This article contributes to existing literature providing a compilation of different entry points of tramp elements in scrap during the recycling process, categorising these element types after the intentionality of their addition and their desirability in the outcome. Although this article adopts a qualitative approach, future research could broaden the scope by incorporating quantitative data (e.g. impurity percentages, material losses, etc.).

## Supplemental Material

sj-docx-1-wmr-10.1177_0734242X251350541 – Supplemental material for Lost in the shuffle: A taxonomy for the accumulation of unwanted elements in steel recycling Supplemental material, sj-docx-1-wmr-10.1177_0734242X251350541 for Lost in the shuffle: A taxonomy for the accumulation of unwanted elements in steel recycling by Beatriz Pérez Horno, Andreas Feldmann and Peter Samuelsson in Waste Management & Research

## Annex

**Annex 1.** Interview guide.

Expertise	Goal	Process/stakeholder	Question	Subquestions/prompts
Technical (furnace operator). Academics/experts in tramp elements in steel.	Knowledge on the chemical composition of scrap and the tramp elements that can be found inside.	Melting/recycling (steel producer)	What elements are commonly present in scrap? (List)	What amount is there from each of these on average in scrap pile?/What quantities for each of these do you handle on average?
				Where do they mainly come from?
				At which stage in the life cycle do they enter?
				What is their purpose? Are they added intentionally or unintentionally?
				In which form? (heterogeneous/homogeneous)
				Were these tramp elements already within the mixture when they got to the scrapyard or are they usually a consequence of the activities of the scrap dealers?
				Are these tramp elements intentionally introduced or are they a consequence of the sorting process?
			Which of these elements do you consider tramp elements?	Why? For what reason are they tramp elements?
				What quantities for each of these do you handle on average?
				Which ones are more problematic and why?
				Are they considered tramp elements in any form or just when they are present in a specific format?
				Where do they come from?/How do they enter the system
			When identifying a tramp element in the mixture, what parameters or characteristics of it are contemplated? (form/composition/properties) and which ones are overlooked? Why?	
			How do you track tramp elements and how often?	Which checklist is followed? European requirements or one of the company’s
				How do you deal with them? Can this be improved?
				What is the decision-making process to decide whether to act on these?
				How rigorous are the auditions/tests conducted to the scrap and its tramp elements?
				How are these documented or notified?
			Which of these tramp elements are given priority?	How do you prioritize their importance and which one is addressed first?/How do you define which one is a priority?
			How do you recover/recycle these materials/elements?	What is the process conducted to extract them?
				Why is it conducted that way?
				Is it always an anthropogenic process or do any of these leave the system by their own means?
				How can each of the tramp elements be recovered under current technologies?
			Are some tramp elements separated before entering the furnace? Which ones and why (not)?	Can any be separated pre-melting?
				Those that can be separated, are they present in any specific form?
			In which form are these tramp elements present in the post-smelted scrap mixture?	Which ones remain in the liquid steel?
				Which ones go to slag and which ones to dust?
Scrapyard and furnace operators	Knowledge on the recycling/melting process		What factors affect your work and the recycling of materials?	What are the major constraints to improve the process?
				What factors require more money, time, expertise, resources, etc.?
			Is the industry facing any boundaries or bottlenecks when extracting these materials?	If so, which ones?
				How are they addessing these currently?
				What are the limitations for doing it that way and not any other?
				What losses do these entail?
				Which ones are more difficult to recover and why?
				Do you recover all or some of them?
			How do you envision perfect recycling?	What are your future improvements?
Purchase manager, scrapyard manager	Knowledge on the products/materials inside the scrap pile delivered from suppliers	Recycling and sorting (Steel producer and scrap dealer)	How much metal scrap do you receive per day?	
			What products end up frequently in your scrap yard?	What materials can be found in each product?
				Is there any material/product that arrives and you can’t deal with and must throw away?
			Where do each of the materials found in the scrap pile come from?	Which Industries?
				Who are your suppliers? How do you choose your suppliers (what are the requirements)?
				What are the specifications/requirements of the scrap you buy?
				Do you import scrap? Where from?
				Does it come from several sources?
Scrapyard manager	Knowledge on scrap grading and categorisation	Recycling and sorting (steel producer and scrap dealer)	What are the KPIs or characteristics that certain piece needs to fulfil to be categorized under a specific grade/container?	What is the composition defined for its categorisation in the recycling centre.
			Which aspects do you first evaluate when you receive the scrap?	Do you track and document where all scrap elements come from? If not, why so?
				Do you evaluate the composition, form or other properties?
				How important do you think the form of the elements present in scrap is?
				Who determines what to look at/evaluate? (EU policies/company)
				How transparent is the supplier with the composition of the scrap?
			What do you immediately do with the scrap once it arrives to the scrapyard?	Do you sort and shred scrap in your infrastructures?
				How do you prepare the scrap before processing it?
				Do you analyse the scrap you receive? If so, how?
			What are the requirements for the products you sell?	
Scrapyard manager. Dismantling, sorting and shredding operators	Knowledge on the sorting and shredding process	Dismantling, sorting and shredding (Scrap dealer)	How do you separate the different products and materials that arrive at your facility?	What are the different decision-making processes involved?
				What works and what are the major constraints to improve the process?
				What factors affect your work and the separation of materials?
				What is the average percentage of a pile that is wrongly sorted?
			Which materials are commonly misallocated? Why?	When sorted automatically (through technology)
				When sorted manually
				Is this misallocation addressed within the company or is it sold mixed?
				How are these reported to the company and to the buyer?
			How do you envision perfect sorting?	What are your future improvements?

**Annex 2.** List of interviewees and their expertise.

Interviewee	Expertise	Type of organisation
A	Ex-manager recycling process	Recycling plant
B	Ex-manager of the production plant	Steel mill
C	Manager of furnace and melting process	Steel mill
D	Manager of scrap yard	Steel mill
E	Head of sales and finances	Steel mill
F	Dismantler	Recycling plant
G	Manager of scrap yard	Steel mill
H	Manager of furnace and melting process	Steel mill
I	Head of sales and finances	Steel mill
J	Manager of scrap yard	Recycling plant/shredder
K	Ex-director of steel mill	Steel mill
L	Researcher	Academia
M	Researcher	Research institute
N	Researcher	Research institute
Group interview A		Steel mill
Group interview B		Steel mill
