# Supplementary material for: Lost in the shuffle: A taxonomy for the accumulation of unwanted elements in steel recycling
Source: Waste Manag Res. 2025 Jul 28;43(12):1962–74. doi: 10.1177/0734242X251350541 (PMC12618735; doi:10.1177/0734242X251350541)
Supplement: sj-docx-1-wmr-10.1177_0734242X251350541 – Supplemental material for Lost in the shuffle: A taxonomy for the accumulation of unwanted elements in steel recycling [file sj-docx-1-wmr-10.1177_0734242X251350541.docx]

**SUMMARY NOTES OF INTERVIEWS**

Due to participants’ preference, the interviews were not audio-recorded and rely on researchers’ note scripts during and immediately after the interview. These summaries here aim to preserve the richness of the interview material while protecting the interviewees’ anonymity (and those of the firms mentioned during the interview).

**Focus group interview A at a steel mill.**

**Attendees: R&D Expert Metallurgy, Sourcing manager, Development engineer Raw Materials, Manager Director Purchasing.**

*The data collection process start with a preliminary focus group interview with a steel mill. It counts with four attendees, whose roles in the company are introduced above. Following this interview, a visit to the scrapyard was conducted, which counts with a breve summary of the observations and conversation with the hosts of the visit.*

Nordic steel producer that specialises in stainless steel and specialty high alloy steel grades. They manufacture secondary steel products with the use of external and internal scrap as well as virgin materials and alloys. Although their will is to transition to a bigger use of scrap in their activities, the speciality and narrow specifications of their products, developed according to customer demands and market needs, still require the readjustment of the formulas with virgin material. In terms of the scrap they employ, 40-50% is internal scrap that comes from their activities within and includes the use of by-products such as slag and dust. Another 40 % is external scrap and the remaining 10-20 % is virgin materials. The proportions differ between different grades, where steel grades with wider tolerances have a bigger share of scrap. The global internal sites perform their sorting before sending the material to the primary steel making facility. The external scrap is purchased from scrap dealers and customers from northern Europe and the global market, who sort and shred the material before their delivery.

The scrapyard in the selected case stocks both, external and internal material, separated according to the groups and grades defined by the company taking the aforementioned specifications into consideration. Moreover, the scrap is divided into two types of raw materials: stainless steel and carbon steel scrap. While stainless steel scrap is kept separated into internal and external, iron scrap is kept together. The composition of these different materials varies between delivery lots, and therefore the scrapyard requires to analyse the composition to be able to produce the product demanded by the customer. Nonetheless, the analysis and treatment of these samples according to the material and their origin:

- Stainless steel scrap.

Due to the various grades of stainless steel and acknowledging the diverse tramp elements that this material can contain the analysis of such a material is necessary. External stainless scrap is typically bought in deliveries of 300-600 tonnes that have a consistent composition, i.e. each sample from any part of the delivery should have the same composition. The analysis is conducted through a sampling process, where a sample batch of scrap metal within the same grade is melted and then examined to determine the chemical composition of the scrap. This will determine not only the tramp elements, but the percentage in which all elements are present in order for the organisation to adjust the composition with the pertaining alloying elements that require addition to reach the product requirements.

- - Internal. Internal scrap is sorted differently in the various sites and therefore the composition is still uncertain. Therefore, this kind of analysis is conducted to be able to produce the required final product. However, the analysis of this is less frequent and simpler, as analysing all scrap coming from the different international sites is time- and cost-consuming.
  - External. In the case of external scrap, the composition of the sample will then be communicated back to the supplier and an arrangement in terms of the purchase will be made. The presence of tramp elements in this type of products varies according to the price of the product, where high-nickel and low-contaminated products tend to command a higher price. However, the presence of some impurities, such as phosphorous seems to be inevitable regardless of the input scrap.
- Carbon steel scrap.
  This type of scrap is analysed less frequently, and its composition is assumed according to the historical knowledge tracked over time (once a year). Moreover, no sampling procedure is performed in this case, as it will only show the tramp elements and no further alloying additions are required for this type of product.

Taking all the above into consideration, the scrap blend contains an array of materials coming from diverse sources and present in many different forms. Secondary material (which counts not only of iron, chromium and nickel as specified but also other elements that derive from coatings, paint and already existing contaminants) is mixed in the furnace. The scrap blend counts with primary material and secondary material that are intentionally added to the furnace and also other unintentional pieces that originate from the different stages in the sorting, shredding and recycling process conducted in internal and external sites.

The expected main product fed into the furnace is the secondary material. In the case of analysis, this is grade 304 Stainless steel scrap. This particular material consists of iron and the specified alloys, chromium and nickel. However, the content of each of these two alloying elements is uncertain and vary from piece to piece. In addition, the piece is likely to also include other elements that derive from potential coatings and paint layers, and some other contaminants that were accumulated during their recycling, or in previous life cycles. Therefore, the composition of the secondary material is ambiguous and fluctuant. Some of the tramp elements are phosphorous, copper, cobalt, tungsten or vanadium.

Moreover, there is the possibility that other steel grades, metal grades or even other material families end up in the 304 blend for the same reasons as exposed above. Examples can be similar steel grades in the 300 series, such as 302 or 316, which can cause the introduction of elements like molybdenum. At the same time, other metal grades can be included, such as steel grades of series 400 or other alloys. This phenomenon increases the presence of unwanted elements in the molten composition. Lastly, some other material families are expected to be found, including I.e. wood or cutting fluids.All these elements are then melted together in the steel making process, where the array of the different elements can be analysed together with the content of each in the new product. Although the causes of the presence of some elements can be clear in some cases, some others such as Phosphorus currently lack a single root cause.

*Following this preliminary focus group interview and site visit, several individual semi-structured interviews took place.*

**Interviewee A - Ex-manager recycling process**

There are different types of scrap flows. Cars, electronics, and municipal waste such as cans and white goods are collected and brought together into the fragmentation stage or shredders. Cars and electronics are a scrap flow that is very specific for the problem of contamination (a significant contributor to contamination). Scrap first goes to dismantlers, who remove economically valuable components like circuit boards (they have gold, silver and other relevant materials), catalysts, batteries, and hazardous like explosives or liquids. Yet, valuable parts such as small electric motors in cars are often left in the vehicles and removed manually after shredding due to cost constraints. The small motors in cars need to be handpicked. This process involves 2 or 3 persons (cost of labour) and are not always located in areas with easy access. The process varies among dismantlers, with some prioritising profit over thorough separation. Dismantlers will sell the processed scrap to the scrap dealers. This scrap is mostly sold by tonnage, so the focus is on maximising the volume of scrap. Thereafter, those dismantlers that get paid by volume might not remove as much elements as those that do not. This means that the extent to which parts are separated depends on the business model and trading contract of the specific dismantler and its buyer (always working towards the highest profit). “If they cannot sell the spare parts, they keep it in the car”. Once scrap reaches the dealer, the mix of scrap from different sources (different dismantlers) and types (e.g. cars, fridges, plastics) is combined on a conveyor belt. All these products are mixed to optimise the process and look for the most cost-effective way. These are then shredded. Manual picking happens after the shredding.

“Every dismantler works in a different way. It is not homogeneous what they do with the material.”

1^st^: collection point, drop the car. 2^nd^: dismantle valuable components with hand-picking. 3^rd^: Shredder (combined operations – flatten cars). They make the cars flat so they can transport as much as possible. They reduce the costs in transportation and they get more money because they sell more tons of scrap. 99% of all cars in flattened. Optimise to get maximum volume in the truck.

*“All the materials and products that are valuable, and with valuable we understand economically valuable, are removed with handpicking. Toxic material is also removed (e.g. the coolants in the fridges). However, they just remove the parts that make sense in terms of time and money. For instance, small motors that are all over cars are not separated beforehand and will end up in the shredder and be removed afterwards when possible. “*

Key sources of contaminants include:

- Increasing the use of alloys and other advanced materials in products leads to contaminants that are difficult to separate from steel during recycling. *“There are more and more alloys in the product by itself. Product complexity is the real problem. Product design is key, if they keep on adding alloys then they are already in the steel and cannot be separated. Most of the materials are already in the steel alloy”.*
- Another challenge is scrap classification. Some scrap is classified as construction material (e.g. used for roads) to meet the recycling targets – EU’s 95% ELV directive. EU directives influence how dismantling is conducted. Particularly for electronics, fridges, and other regulated materials. For instance, fridges undergo vacuum shredding (controlled environment) to capture Freon. Some products have their own specific organisation (e.g. those that take care of electronics or batteries).
- There is a conflict of interests between the different actors involved. Operations are always money-driven and look for the most cost-effective way of working. For instance, shredders could lower the content of copper. You hand pick a lot of material, but there needs to be a balance. The goal is to optimise the output. Slow down the process could help in taking out more material, but it will cost more money “*Practically you can shred to finer size distribution with better separation but at a higher cost and lower productivity*”. There was one recycling organisation that tried to have different flows in the shredder but they found out that their productivity went down. The most cost-effective is to just mix everything. Moreover, doing so would require higher maintenance of the machines. *“It could be possible to slow down the shredding process and get better separation through density, but that would entail a loss of productivity. All strategies and actions are led by what makes financial sense and productivity sense.”*
- The separation of certain material depends on its value. There needs to be value created for certain materials so that new businesses can come out of this and somebody takes care of it. This is the case of plastics. Need to make a business and economic value out of it (develop positive value for the fraction). Hence, some of the plastics are removed pre-shredding for energy recovery. Another factor is the amount. If the material is present in small amounts it is not cost-efficient to remove it. Lastly, some materials are covered (you can’t see them). This happens a lot with copper fibre.
- Storage is a very big problem now a days in terms of keeping materials separated from one another. If you split the material, there needs to be enough space (space constraint in the yards). Thus, scrapyards cannot handle/afford having several piles of different materials. Consequently, all products are put together to optimise operations. Grades are less specific and piles are combined.
- Transport is another big issue in the cost calculations. This is why recyclers are organised geographically in Sweden. Each recycler takes care of a selected area in their surroundings. There is no competition between them, each get the municipal waste from their area.

Material examples: phosphorous (not sure where it comes in, cannot be removed from stainless steel as all chromium would come out); Zink (mostly from coatings yet it is not a problem as it ends up in the filters in the EAF as dust).

**Interviewee B - Category leader for Raw Material purchase since 2006. Before in charge of the optimisation of the scrap loading, business control.**

The inflows of scrap (3 different: scrap that is more or less direct, from own production or from their manufacturers. Scrap can come immediately (5-10months later get it back to the value chain); white goods and cars will come back in some way sorted or unsorted in sometime within 7-15 years; old steel that are in the infrastructure that will arrive in around 70 years. Direct material in a shorter time than a year they know what they can do and achieve. When it comes to second layer of inflows (white goods and automobiles), they need to ask the producer: how is the product designed for recyclability. Is it easy to remove the copper cables? If it is easy to remove them they won’t have problems with copper. Copper is the main issue, the main source. You usually have copper wires everywhere in a car (e.g. wiring or electrical motors). The wiring is an issue that you can solve with better separation at the shredder. Motors and rotors are not that easy. The magnet will not separate these appropriately. Separation with magnets or other robotic arms has been more or less a failure so far. LIBS system – very slow operation and focus on high value scrap, scrap from aerospace. The technology is there but there is a breaking point. Other trade-offs are the problem, not the technology, it is more of an economic decision.

Different steel mills have different demands. Molybdenum and nickel are tramp elements for some plants but needed for some others. Copper is a major problem for practically all steel producers. There are a couple of issues with phosphorous, you can have it in some iron products and you have it in the dirt. If you have dirt from the ground in the iron. From the car industry some phosphorous content can go in the steel (e.g. a lubricant from the automotive industry). Surface treatments with phosphorous. White goods have titanium content in the white paint, an issue for some of their plants. Titanium will end up in the slag.

The different steel mills in Sweden cooperate much easier than the scrap dealers. The scrap dealers are not happy to talk to their suppliers. No openness at all. The cars are collected by car scrappers that sell the scrap cars as spare parts or scrap suppliers. The scrap car suppliers, how they treat copper is an interesting issue. There are rumours now that the car scrappers are pressing the car and the copper cables get inside. It is more economical for them to take out the cables or to compress them? Do they get paid properly for the copper? Talk to a car scrapper to understand them.

Scrap sorting system internally could also be wrong. High nickel content pushes the steel out of speck. If you get molybdenum and nickel that is still in speck, that is a problem. The scrap dealers know this content and they try to sell it to others. They have several contracts. Pay 15% of the nickel price in that nickel content. Try to lead/incentivise recyclers to sell low alloyed scrap to the steel mills.

Need to reduce the demands of copper content in the scrap they buy. In 15 years time they will have a problem in copper content if they accept higher limit on copper. “*30% of the copper content melted in the steel is originated from copper cables that is not sorted in the scrap*”. They want them to separate this. Antimony is always a hassle to understand where it comes from, railway cars or big old trucks. Plastic can also get in the scrap. They made a test several years ago with windmills and they did magnetic separation: non-magnetic material and got all the plastics, not 100%. Every delivery was inspected by them and approved. The worst delivery had 6% of plastics impossible to detect. 10 tests, 5 per shredder suppliers (2). Normally it was below <1%. Two of them were 6%. When they checked the material it was impossible to detect it (with visual inspection).

**Interviewee C – Manager of the furnace and melting process in steel mill**

The scrap recipe that arrives at the furnace is already sorted and selected based on the chemical composition specifications of the intended outcome. To make this recipe, a mix of different scrap grades and different scrap shapes from each grade are combined. The type of products that are charged in the basket that is to be loaded in the furnace is critical, as we want to keep a good balance between the scrap shapes, lengths and weights to optimize the melting process. In an ideal scenario, the basket contains light, medium and heavy weight scrap. The heavy scrap is charged at the bottom of the furnace and the light scrap is left at the top so the electrodes get down quickly. Most heavy scrap comes from our own mills, acknowledging that around 40% of the scrap at the scrapyard is internal scrap from all the mills owned by us. Hence, the scrap basket needs to be as similar as possible from one another, to have a similar charge.

Although scrap is selectively analysed with laser “guns”, there are some elements in the scrap pile that are not intended to be there. A problem and a real danger this time of the year is ice. When ice gets in the arc furnace, it can cause explosions. On the other hand, after melted, there are some elements that one can find in the liquid steel. An example is phosphorous, from which we do not know its origin (where it comes from). Phosphorous can be removed from carbon steel but not from stainless steel, entailing a problem for this last material. Nickel is another metal that could be harmful in the case of carbon steel, as it cannot be removed from this. Putting nickel scrap into a low alloy steel is problematic as you can’t oxidise it. A bigger problem are oxides. Magnesium oxide (around 20% is the optimal wanted) and Calcium oxide are added in the process. The appearance of other oxides complicate the process as they reduce the amount of magnesium oxide in the melt. These first two (magnesium- and calcium oxide) often end up in slag or dust after the scrap is melted. But there are sometimes a type of material which is a combination of steel and slag. In these cases, this material is processed and steel is removed from slag. However, this steel tends to retain some of the oxides. This steel with oxides is fed back into the furnace, entailing a problem for the new material as these oxides get trapped in the new recycled material. *“Although it is not our case, as most of the products they receive are not painted, painting and coatings are also a problem in terms of contamination and accumulation of unwanted elements in the chemical composition*.”

Slag and dust are needed in the processes. However, neither of those are wanted for the mill as “*they are basically lost*”. Around 8% of the metal that you feed in the furnace does not end up in steel, which means that those elements get lost. Some examples are: iron, silicon, manganese, titanium, chromium, vanadium. Besides the loss, the plant needs to take care of the generated slag and dust. The dust is sent away to the south of Sweden where they recover some materials that are afterwards brought back to the operational site. Slag is accumulated in the plant and treated by an external partner. Some oxides can be recovered from slag and treated to get the metal ore. However, this is not done. Currently slag is stored. Either way, there is an ongoing project to use this as a by-product and give it a further use.

Some characteristics that are relevant for a productive melting of the scrap are: weight, shape and length. Too big pieces can entail problems, but too small ones too. However, the plant still uses big material. This is because it is not economically attractive to cut internal ingots for instance, and these are also charged as material. Therefore you need a balance and to fill up the furnace in a good way avoiding cavities. For this reason not all the purchased scrap is shredded, the scrapyard has a combination of big, long materials and shredded materials. We do not have shredders. Besides, too much shred scrap is not good because it gets stuck in the walls and therefore it is not beneficial to charge 100% shredded scrap. Another product that can harm the melting process are coils. These can sometimes be difficult to melt as there are thin layers of air between every coil and every layers melts once at a time. There has been cases where after the melting process, the coil is still there and has not been diluted.

Scrap density is very important for energy consumption. Solid, big pieces take longer time to melt. Given that the purpose of the mill is to melt the scrap at the lowest cost and the highest productivity (less energy, less electrodes, less refractory), there is a need to optimize and balance the input material that is fed in the furnace (it requires a mix of different scrap grades and sizes). However, sometimes even the same steel grade or recipe can show different results in energy consumption. There is a recent example where the same grade in two different hears consumed a different amount of energy, as one had lighter material than the other. However, this density and weight only affects the energy consumption, productivity and costs, and does not affect the end product contamination. In terms of challenges for this optimization, as said the shape of the scrap and the amount of oxides are the greatest challenges. Overloading or having heavy pieces at the top of the pile can also reduce the productivity.

Due to all the above, having a knowledge of the exact scrap composition is very relevant. Else, you can have problems afterwards. Sometimes you need to take away 5-10 tons of scrap to introduce some alloys that compensate the composition.

**Interviewee D – Manager of the scrapyard in steel mill**

Our company handles internal and external scrap. Heavy weight scrap comes mostly from internal scrap, while external scrap is in its majority low density plate scrap and sheet metal. External scrap can be separated in the following: (1) Stainless (low density), (2) iron scrap (there are 10 different types with different composition bought based on past experience. These vary a little bit more, sheet metal, special iron scrap), (3) chromium scrap (iron alloy with chromium), and (4) high-nickel scrap (blends rather than homogeneous scrap). Internal scrap includes ingots, billets, bar cuttings, strip scrap, etc. Categories of internal scrap: Residual products is about 15% of internal material product; bulk groups (majority of the volume, easier to separate as they are large), premium groups. Mainly residuals from processing material and turnings. In terms of scrap piles, internal scrap is mainly present in just one pile, while external scrap can be divided in different formats: one pile of solid scrap and one pile of lower density scrap. Whether a certain group is divided or not is mainly defined by the volume of the scrap. Dupplex, low alloys and scrap containing molybdenum tend to be the biggest volume of scrap. Sometimes new steel grades and new internal scrap groups can be created. However, the first one takes a lot of time to achieve a new production volume and therefore is not frequently occurring. The second happens more regularly, especially with speciality groups.

The purpose of introducing internal scrap with external scrap in the furnace is two-fold: on the one hand, it is needed to include heavy scrap with light scrap, and on the other, it is a way to meet the composition in scrap with difference tolerance contamination levels. Some “contaminated” groups are: higher-nickel and low in phosphorous (which are very clean), lower in cobalt (which are the really really clean ones and with the highest price), and some materials that can tolerate contamination. There is a scrap group with Titanium and another with Niobium. Although most of these elements seem to be wanted in these specific products, there are some elements that are contaminants for all groups, such as phosphorous. These additional elements are not removed from the chemical composition as it is a very costly process.

Slag is needed in the metallurgical process and its generation is optimised in the process. You don’t want it to be expensive, don’t want to add more process materials or oxidise valuable elements. Slag is extracted, cooled down and then it undergoes a deslagging process. Here iron is removed to be used again in another melt. With wet-grinding the remaining parts are recovered and break down into light oxides). There is always a trade-off between achieving the desired product and the cost. Filtered dust, which is often a combination of light pieces, is not wanted. This is put into bags and sent to an external contractor to reduce the material, which is a very expensive process. However, it is necessary to get the valuable metals back (mostly oxides). The slurry is sent to landfill (chromium oxide mostly ends up here). The composition of both of this is not unexpected, but counts with elements that end up in the metal.

*“Among the challenges that we are facing with external scrap, they mostly are related to the availability of affordable iron scrap, which is clean from contaminants*.” Pure iron is needed for low-alloy grade, while for stainless material this is not a problem as you can always add virgin chromium and nickel. Low-alloyed material is very sensitive to contamination and there is not an economic option to make it more clean. In terms of internal scrap, the sorting process is what is more problematic. As it comes from many different places and the sorting is conducted in different ways in each of these, material gets mixed. There is no traceability and therefore material can spill over from one pile to the next, or mixing can occur in the cutting process.

The goal is to maximise the amount and quantity of premium groups as possible, as it is a financial loss to have less valuable groups. For this there needs to be a better separation throughout the chain. This is easier with industrial scrap than end-use post consumer scrap (as this is all mixed up). However, scrap dealers could increase cooperation with industries that buy the scrap to generate more information on the composition of the scrap and certify that when they sell it.

**Interviewee E – Head of sales and finances in steel mill**

**Interviewee F - Dismantler in recycling plant**

The recycling process looks at follows (must be noted that production scrap is already very homogeneous and there for not much sorting is conducted, just manual sorting after crushing to make sure there are not too big pieces and these are clean):

1. The end-of-life products are collected either by the municipality or by small companies. Smaller companies tend to remove some material manually, while municipal waste and medium and big collectors usually approaches the recycling centres without any previous analysis. This collected material is usually a big mix of products which composition varies depending on where it comes from and the grading system of the company.
2. Once it reaches the recycling plant, the products are scanned to make sure there is no radioactive material or hazardous material. It is not very common to find radioactive material but when it happens it is sent back to the supplier. From 0.5 million ton of scrap that reaches the plant, a big percentage of the material does not belong there. This is because there is not economic value in performing handpicking in advance. Hence, one can find tires from bicycles, interiors from cars, etc.
3. The process at the recycling centre follows a lot of systems to make sure that every fraction is as homogeneous as possible.
   1. The arrived material enters a 30-40m conveyer which moves the material upwards, so it can after fall down to a crusher in a perpendicular way. The input material that enters the crusher should have a big enough volume/mass, therefore additions products are put on top of cars. The biggest the mass of the fed material the more even the output is. Hence the lines are usually kept evenly full.
   2. Once it enters the crusher, the products fall into a zig-zag system where gravitational separation takes place. Additionally, air is blown upwards through this zig-zag to get rid of light material such as car fluff. Besides, a shaker shakes the material to detach all products and materials as much as possible.
   3. Inside the crusher there is a rotor with hammers spins that crushes everything down to pieces. However, it can also happen that due to the speed of the hammers, some materials get molten/blended together at this stage.
   4. Finally, the shredded material goes through a magnetic separation where ferrous and non-ferrous are separated, for instance, metal wires and light metal is removed. There is a following magnet (Steiner magnet) which has a higher voltage and helps remove aluminium (which can come from cans, e.g.). All material that is separated by the magnets does not go to the final products and re-enter the process again (they are fed again into the crusher). However, when you have a big mass of products there are high chances that some material can end up in the wrong place. Non-ferrous go again through another magnet belt that takes metals away in a separate conveyor.
   5. Hand sorting is done at the end as the final ferrous product cannot be too big. These are fed back again into the process. In occasional events, the final outcome is analysed.
   6. Dust collection is also performed and it is treated separately and sent to landfill or incineration.

E40 is the most common product for municipality metals, it contains rails, cars, pipes, galvanized steel, etc. E1 is formed mainly by iron rims and material that is 3mm, this cannot go through the crusher as they can ruin the hammers. E2 is production scrap thickness. And E3 is metal scrap over 1.5m. These last one needs to be cut into smaller pieces.

The most challenging aspect for these metal recycling are the additives, the way in which different materials are assembled. If ferrous and non-ferrous are attached very hard in the design, the ferrous material will win in the sorting and therefore there will be some non-ferrous material in the ferrous scrap. Welding ferrous and non-ferrous is very problematic, however it is not very common and these are usually assembled with screws. However, if these screws are made from brass, you lose the brass as it will follow the metal in the process. Anything attached to the iron will be picked as iron in the sorting system. Aluminium 4C is an example of a very contaminated material, contaminated with steel. Other materials, besides metals, are generally hand sorted and do not end up in the mix, such as plastic. This is also because plastic burns quicker than iron, so chemical materials attached to steel won’t end up in the secondary raw material as it will melt up. Hence, gluing and welding are critical points to take into consideration.

**Focus group interview B at a steel mill.**

**Attendees: Manager of the scrap yard in steel mill and manager of furnace and melting process in steel mill.** Involved in the use and analysis of scrap since it arrives at the facilities until it reachers the converter.

They buy internal (45-50%) and external scrap, but the latter is a very small percentage. The main residual elements that can be found in these are copper, nickel, molybdenum, tin. Internal scrap includes more than 40 different grades in which this is separated. External scrap follows the European grading system. They currently not buy a lot of post-consumer scrap. External scrap is new scrap from OEMs with no big deviation in composition. 700 thousand tons of E6 and E8 and some grades E2. E2 scrap has higher residuals. Post-consumer scrap has 92% iron content in comparison to the 98% in the rest of the products. Residual elements are also not interesting has they reduce the amount of iron in a product and therefore entail that more iron needs to be added. Hence, they are not a loss by themselves but they also entail a loss of iron per product. E2 and E3 (post-consumer) are stored as 1 group used for grades with higher tolerances. This has a lot of air in between, lower density.

After the melting process, a sampling and analysis of the steel and the slag is performed. Hot metal analysis. Once a year all this data is put together to update the database from the scrap grade. The increase in copper stopped because of a better sorting process. There is an increase of molybdenum because of additional steel grades in the new years (complex alloying mixtures). There is a need to provide high quality scrap automotive surface., high strength steels and this is often not possible with the use of post-consumer scrap. Consumer scrap is low in density (a lot of volume but not weight), which is also not wanted for the recycling of material and therefore the amount of volume they can purchase is limited. Internal scrap is not 100% certain of what it contains as it can also include demolition scrap from facilities. Limit chromium input as most goes to slag. Some dangerous coatings can include Lead (Pb) for corrosion resistance and in old pipping. This goes to gases and it is dangerous. 20 kg of dust per ton of steel (iron and zinc oxide) is produced. This goes back to the steel process and the rest is sent to recycling facilities to get back the zinc.

The scrap recipe combines different grades which are chosen through an optimisation program that determines the cheapest scrap mixture that meets the tolerances and demand. The recipe includes a mix of scrap grades within the tolerances.

Slag is mainly dumped and its treatment comes with costs. The more slag it is produced, the more scrap is needed per heat. It is recycled to get iron back and the rest is sent to internal landfills and will after be exported to other countries. In slag, iron is lost. The more phosphorous diluted with slag produces higher slag amounts. Having more phosphorous makes them produce more slag, cause they have to add more iron to dilute it. Phosphorous oxide between 1-2%. Some iron ores in the converter include alloying elements (that is how phosphorous gets in). The post-consumer scrap coming from the scrap dealers can include a lot of non-wanted material. *“Scrap dealers get ride of scrap they don´t want and fill it in the composition of the scrap delivered. Hence, the people receiving the material have to check the loadings”*. Scrap catalogue – the scrap grades are separated and defined after the thickness, whether it is new or old scrap, a magnetic check. Aluminium is sorted out. Sometimes one can find liquid in radiators which is very dangerous as it can provoke explosions. Scrap dealers or suppliers are selected for their price. The ores are mostly coming from Ukraine, South Africa and Brazil. “Everything depends on the cost in the end”, the total costs. Prize per ton of ore is around 100€ and per ton of scrap 350€. Scrap is more expensive and also is less pure. Also the process of producing secondary scrap is higher than the one to produce primary scrap, and it entails additional time. This hinders them from using more scrap. BOF is still cheap as a process. The same material is present in different forms.

The scrapyard is designed in a way in which each field includes a different grade. Internal alloyed, internal unalloyed, external. Regular checks are performed in the scrap to make sure that the products in a scrap pile are actually matching the grade. Logistics are the biggest challenge (rail system), which is to be increased with the transition of one BoF to EAF, as this means that more scrap needs to fit in the installations. Capacity of the scrap yard, installing an EAF makes the need to increase the scrapyard and te use of post-consumer scrap, which will depend on the price.

Although shredded material is very little brought and used. HBI is also limited, lose time and iron in the process. HBI is avoided as much as possible, it is very expensive. Pig ion obtained if the process runs too slow, here sulphur is a problem from the desulphurisation in the steel plant. Virgin material at EAF bought from BOD with high content of sulphur if you can handle sulphur in the mix, as it is a cheap material. Silicon content in electric motors steel, it affects heat balances and lowers iron content which entails additional energy, however it is handable. Important to know the composition, however no information is received from the suppliers.

Internal scrap sometimes need to go through a cutting process to be able to fit in the furnace. Size is important. The max size is 1.5 x 0.5 x 0.5 (3-4 tonnes). EAF needs even smaller sizes. This change will also entail adding new scrap for manufacturing and the use of external scrap, different scrap balance. However, quality will change. Some products that end up in the scrap pile and can impose a problem are: electrical motors, pipes (if they are pressed together they can include water or liquid) and fittings, bearings (cage, which include bronze), electronic scrap, springs. The way materials are assembled is a critical factor in the recycling.

**Interviewee I – Head of sales and finances in steel mill.** Purchasing manager, involved in the development of new business strategies with scrap dealers and the new EAF.

800 thousand tons of scrap now in comparison with the 2.2 million tons they will need to purchase for the EAF. Market is changing a lot. Mostly pre-consumer scrap. Currently 20 million tons of scrap is exported mainly to Turkey. Quality issues are hindering them from changing to EAF and the costs. *“You cannot obtain surface car quality with scrap.”*

Storing scrap is a main issue (stock), therefore they need big stock yards for buffer. The strategy is to absorb the scrap dealers and create scrapyard hubs in their facilities. Around 3 hubs are in their construction to store the necessary increased scrap. They need better input material from the dismantler to the recycler/dealer. Scrap hub logistics centres (3 in Austria) - digitalised information and tracing of the scrap (track quantity, type, etc.). It is the first steel mill in Europe doing things. Scrap dealers working mostly only for them. Scrap dealers become a logistics provider. USA steel mills have their own scrap dealers. The goal is to have more influence in the development of the dealers, their sorting technology, logistics, and a good way to start in other countries. They ensure the dealers invest in innovation and technology and increase the quality of the material and the storage capacity.

OEMs scrap – selling steel and buying scrap contracts. Sell green steel to the scrap suppliers.

E40 is shredded material which is manually sorted. This is very expensive but has the quality of almost E2.

Closed shredding process and machines are an option. LIBS analysis only makes sense in shredded material. Green stamp in the product even if it is produced at BOF.

Biggest challenges: product design (design for recycling) and the need to develop new ways of performing at the shredders, which are always doing the same. Challenge to hold scrap in Europe and not export it. Need for better policy specifications and have a better certainty in the political situation.

Key takeaways are:

- They are slowly implementing secondary production. However, it is not their main goal as it comes at the expense of the quality of the material and the process and input material is more expensive.
- When using external scrap it is very important to know the composition, which is currently very uncertain. Therefore they see the need to absorb the scrap dealer business and perform them themselves, having a better control of their work and involving them in the improvement and investment of better separation processes.
- In terms of input material, the quality and purity is a critical factor. Also the density of the product. For this two reasons, external scrap is not seen as an advantage, where considering the latter, the material is very light.
- Residual elements are not only a loss and a contamination problem per se, but they also entail that the amount of iron in a product is also lower, hence it is seen as an iron loss.
  - Some residual elements that are not wanted: copper (although it is less present thanks to slight improvements in the sorting process), molybdenum (coming from the alloying complexity of products), silicon from electric motor steels (although this can be handled), lead (added for corrosion resistance and in pipping), phosphorous (coming from iron ores that include some alloying elements). Bronze coming from bearings.
- Scrap can be separated into new and old scrap. Where the latter impose a lower certainty of the material. Different levels of certainty and contamination in scrap.
- Current sorting and shredding processes are not very accurate and the information shared between dealers and mills is very poor. Hence, there is a need to perform checks and analysis at the mills.
- Logistics and scrapyard capacity are the main challenges at the mills.
- Shredded material is just bought and “wanted” in small amounts. Importance of size and density in whether a material is wanted (affects the volume of material bought in these characteristics or specifications).
- The product design and the way in which materials are assembled is a determinant factor affecting the recycling process. Also the sorting process negatively affects the recoverability of resources.

**Interviewee J – Manager of the scrap yard in recycling plant/shredder**

The shredding process is conducted with a hammer that works at 1400 kW. The shredder handles 80.000 tonnes of metal per year, from which 11.000-12.000 are from the automotive industry. They also handle cans, steel sheets, washing machines (very complex), etc. Old cars are the biggest fraction. This, together with electric products, household scrap, sheet scrap, municipal scrap and metallic packaging go through a separation manual pre-treatment where glass, tires, wheel rims (steel and aluminium), pb batteries, fuel/gas and oil are separated/cleaned from the product. Here all the hazardous or sellable products are removed. Now they want to start separating plastic to meet the new market and policy requirements. The remaining scrap goes to the shredder. Here dust is removed together with light fractions (plastic). The remaining goes to a magnetic separation where the ferrous and non-ferrous get separated. Here the extracted fractions are steel scrap E40, steel cans, and copper coils and windings. there is some handpicking to make sure there is not copper, aluminium or unwanted material in the stream. This then goes to a screening process where this material <10 is handled further. Lastly, an eddy current process separates the following fractions: Al cans, non-ferrous scrap in two different sizes (fine and big), and rubber in two sizes (fine and big).

It is no problem for the shredder to have a mix of different material as long as the thickness is below 5mm. The important thing is to make sure no problematic material enters the shredder. The most important aspect they are challenging and caring about now is the quality of the material. They have to make sure there is no copper, high alloys steel (vanadium, nickel alloys), aluminium. They need to improve the quality as the industry is using more and more scrap now. The requirements are getting stricter. The quality needs to be ensured earlier in the process. How can they guarantee the composition? They need to have a better idea of what they get.

The shredder cannot be optimised, they are getting a new one but the process will be the same, there is nothing to improve there. However, what needs to be improved is the sorting and separation before feeding the material to the shredder (LIBS?). Hence, there is the need to develop technologies that can identify and analyse the composition of the material they receive. Hence, they are facing a technical challenge, as the information is very low. Their suppliers also don´t know what it is in there. It is very complex to know the composition of the post-consumer scrap. The real problem is the material that comes from the municipalities, the automobiles, household scrap and steel sheets are more or less known.

The grades of scrap are very broad and wide. There are 6 shredders in Austria. All their output is sold to the mills or to the recycling plants to process non-ferrous. 85-90% of this other material is aluminium. They need to improve the quality of the steel scrap they are selling and make sure it does not have copper or aluminium. Better separation (automatic). The old automobiles vary in composition. This are 10-15% of the input material. They receive material from municipal incineration plants. The material they get depends on the market, they cannot choose what they get.

The shredder can only receive 120 tonnes of material max. Copper comes from cables, wires, electrical motors, machines, small motors. Small motors go through the shredder together with cables but they want to hand sort them before in the future. Aluminium in cars comes from levers and housing of gears, motors, etc. Few go to the steel, but this can happen when they get mashed/crushed together in the hammers. They never get to melt together. How the product is assembled impacts the possibilities of sorting. There is the need to construct designs for better separation. They need the process to be more automatic even though the quality is reached better through manual processes. This is because the job itself is not very appealing so cannot be done manually. It is not feasible to do everything manually. However, the human eyes can differentiate better the different materials. They want to perform another manual sorting after the magnetic separation.

There can be aluminium or plastic in the output material. This is because in the shredder the hammer crushes everything together and materials get entangled (meatballs). What is critical is to dismantle better before the shredder and to know the composition of the input material better. Try to get the composition from the ones that deliver the fraction. It was not done before as there was no demand to get high quality but now these exists and the specifications are more demanding. Consequently, the price on the output will have to increase. Suppliers don´t know what is in there, postconsumer scrap is difficult. They will need to perform analysis of the input material. The shredder develops a definition of the quality per client and product based on historical data, on their experience. Is it their job to do it?

The shredders work on demand (reactive). They change only when they perceive external pressures. There is no incentives or motivation to change to improve or to get new market streams. The shredding process is so brutal that almost all paintings and coatings get out.

**Interviewee K – Ex-director of steel mill**

The specifications are what guides the mixing. Dilute the elements - how can you use these materials (trace material). We only differentiate big groups/grades. Important to know which alloying elements are there and how to use them. There are 7 or 8 different steel types in a car. TRIP steel contains manganese in high quantities but can´t be used in standard steel.

We tend to invent new material, variety of alloying compositions, with advantages against competitors. “It is better to have lower types and better requirements. Focus on few materials that can fulfil X applications”. Need to consider the trade-offs of the different materials. Look at the whole – the best solution for the whole life-cycle. Understand and design planning the dismantling. New business approach. Mind set is very important in the system; always thinking of growing and producing more.

Addressing the accumulation of these at the sorting stage is already too late. The optimal would be to conduct sophisticated disassembly before shredding. However, it is too costly to do it, it is cheaper to use everything as construction, but it would create a lot of opportunities. The high tolerances in scrap for construction allows steel mills to always have a business case, sell the product. High quality (special) products are more challenging.

Geometry sometimes hinder reuse. Melt and produce a new material in new shapes. Most trace elements come from previous alloying elements that are not wanted anymore. Copper wires are challenging as you can´t reduce copper from steel. *“Next time the secondary steel already has copper, once you recycle it 20 times, the copper content is too high”*. Antimony we don´t know where it comes from. Tin from coatings and zinc too. They both are in dust but some of it can remain in steel (part per million). Molybdenum comes from mixing other alloys.

Most specifications are done based on the use of iron from iron ore (pure) from BOF. But now with EAF using different trace elements that influence the result it is more complex to meet those specifications. The optimal would be to have lowed grade options, simpler/standard chemical compositions. Yet, it is not possible to change specifications and policies, nobody is going to do it as it takes a lot of time and effort to change them.

**Interviewee L – Researcher in academia (involved in analysing tramp elements in scrap)**

There were some products that are wanted in the product but not desired anymore.

Contamination:

- Lubricants and paint part of the product but not part of the steel.
- Can happen at 3 different stages, which vary on the material:
  - During the production
  - During the usage: you can´t control the contamination during the usage of the product in consumer goods.
  - During waste management: shredder hammers material together and this cannot be separated easily anymore.

There is a need to focus on product and design and in the product´s waste management. See where you can do something. Need to bring together designers, producers and waste managers.

There is no system in place to make materials 100% recyclable, you have to choose a way to go. Plastics are not really a problem. For instance, there are many paintings that are polymeric and most of it will evaporate or oxidise. Zinc coating, copper from copper wires (small parts attached to something), not full knowledge where it comes from. Aluminium not in such a high amount as it gets separated through magnetic separation.

Sorting is not a critical step but how you process the material. Cutting shredder. Magnetic sorting works well, the problem is when it gets crushed and then gets entangled. Construction debris can´t be separated properly because of its size and weight. Material itself is very alloyed because the requirements are not that high. Loaded with tramp elements. Deconstruct buildings and separate the materials at the construction site, after it goes to scrap dealers. Control for the process. High amounts of waste flow to have a system where you buy the recycled material recovered from your own products.

Most tramp elements are not recovered although they are seen as a problem. This is because the amount is not that big. Sampling is not worthy, you don´t know the composition, knowledge is a problem. For every metal you need your own process. Better to target a material that is in there in an amount that is interesting to recover and then set a system for it. Maybe there is the need for a new actor or business that takes care of this. Most elements are solved in a different system, when you get them all together then you still need to separated them in a different additional step. Not all tramp elements are easily extracted by solving. Recovery of the material is a process that is not a skill of the steel mills, a different process.

Phosphorous is a tramp element but there is a process to get it out. It mainly comes from the ore, but not known where it comes from in the scrap. Virgin material with high share of it.

Interesting characteristics for separation:

- Composition
  - Colour and appearance: see what they are by appearance
  - Form: flat, long
  - Density of the heap: give an idea of the forms.
- Where it comes from, what it went through

**Interviewee M – Researcher in a metal Research Institute.**

Optimised use of raw materials in terms of technical efficiency. Concerns on how to drive the process. The time, temperature and additives to transfer the tramp elements into the slag and not into the steel. The slag and the dust are partially recovered afterwards (f.e. zinc, vanadium, manganese, chromium). However, this depends on legislation. In Austria, slag cannot be used in construction but in Germany, one can use slag from EAF in road construction. If we want to lower the share that is landfilled, how many elements are allowed in slag? If regulations get stricter it won’t be possible to use slag as it is right now, needs further processes. Today slag is mainly landfilled. *“Recovery is not yet a thing in Austria, as it is cheaper to landfill”*.

Treat dust and slag to recover the iron fraction. Scrap recycling is new. The increase use of scrap is a problem for the durance, as some elements are difficult to recover, included in the steel mattress. Sinter is heterogeneous and sintering is performed to obtain certain properties and quality. “*Treat slag to get at least the iron”*.

Batteries introduce new elements: manganese, chromium, vanadium. Phosphorous is a critical raw material gaining importance because of batteries. From electrodes batteries in the future, but mainly from iron ore. Not known where it comes from in the scrap. Main process for recovery: melting and reduction, solving. Pure metallurgical, you obtain metal alloyed and mineral slag (residual fraction), which is used as cement. Tramp elements: manganese, sulphur, phosphorous. Alloying elements from electronic waste (steel alloys): manganese, vanadium. They have special properties. What is in the scrap is a problem for steel mills. Heterogeneous material. How is it analysed? Lack of technologies to better know what is in there.

Difficult to characterise scrap: not possible to do everything automatic. Scrap processor needs to work with costs: it is expensive to analyse manually. Steel producers often rely on what they get from recyclers, they don´t have resources to characterise. Different sorting standards in every part of the world and organisation: scrap comes in a more heterogeneous way. Manual operations for high quality products.

Size of scrap pieces (sometimes cutting pre-processing is needed) and the volume –< Geometry. Charging baskets, basket should be as full as possible with the lowest amount of air.

Getting high quality scrap is a problem. Giving that the share of EAF will increase, this is a major area of concern. DRI plant – high grade iron ore (60% iron). How to get scrap cleaner? Producers with different bins- company´s who produce scrap need to make more efforts in sorting. Deliver a better separated material to pre-processors. Better analysis at pre-processors (optical systems, spectroscopic systems, combination of LIBS and XRF). Not many developments to be done in shredder, detect optically at this speed is a problem. Steel producers depend on trust. All need to collaborate.

Deliver the product demanded optimising the costs. Economic: who pays/invests in changes to become “better”? Challenge for collaboration. Different interests: scrap dealers – least effort with lowest costs and highest price – and scrap recyclers (mills) - better quality with lower price. EOL scrap is not much lower than new scrap. Developing countries demand “whatever quality of scrap”. Price market is one of the most important factors. Product demand (product portfolio) is also very relevant in terms of how much scrap is recycled. In Austria 80% of demand is quality steel. What quality is demanded determined the use of scrap share. Import of scrap.

TRS project – shredding. XRF and LIBS after shredding: Improve scrap pre-sorting. Manual separation: more costs (scrap pre-processors want to be as much efficient as possible), working conditions difficult to get employees). Tramp elements affect on properties and quality (f-e- strengths).

**Interviewee N – Researcher in a Research Institute, expert in recycling metal.**

Material connections: gluing, painting- break or not when you shred them?

Base material plus that superficial treatment goes out. If you shred the computer, when you shred you have particles liberated. Reducing the particle size, you get particles with different compositions from the same product. Larger number of particles, but you get mixed ones. Sorting through density, colour. Mixed ones can go either way, always bring in impurities. Allowable amount of impurity level that makes the process economically feasible. Size reduction, sorting, size reduction, sorting. The key part is starting from the end – smelters that can take different kinds of scrap with limitations on what impurities they can remove and how many impurities they can handle. What they can refine. How can we sort and shred differently to optimise the process before? How can I redesign and change my product? Material characteristics that affect that? Compatibility recycling processes. How are they connected together? Some assemblies do not enable the liberation. Screws and bolts, it you shred they break in a different way. Materials behave in a different way. Some material combinations can be recycled together: different steel alloy families. You can do that already in your product. Reduce the number of alloys in the product.

Copper wires and boards. Why do you sell it? What is the regulation (legal requirement)? Being further recycled has a cost. Some companies take the big chips they are rich in critical metals and they sell it for products. They don’t go through the normal process. Take them out all the way at the beginning and sell them before hand. How do you create value through the process?

Logistic purposes in the sizing of the products and reduction. Increase the density. Shredding of circuit boards. Every time you size reduce you create a distribution of particles and create dust. Depends on what your product is made of they become dust (e.g. ceramics). Materials that comes out at the end has lost a lot of the value in dust. Galvanised zinc – zinc recovery in dust. Not yet everything is recovered. Technology challenges and economics. Do we have recovery processes in place?

A lot is determined in the design of the product. E.g. Fairphone case is very interesting compared to other mobiles. Replace the modules. Place the components in a different way than normal. Reduce the amount of size reduction and sorting process. It is an expensive process. Everything that you can change in the design makes sense. Things are not designed to be recycled. Economic challenges – return logistics, raw materials are very cheap. Recycling is very expensive, you need policy support. Aluminium is over engineered. Some things we use because we can and not because it is necessary. Challenge in recycling: primary materials becoming purer and purer. Tailor-made composition for some aluminium companies. Inclusions in the alloys that improve surface. Are we valuing enough end-of-life products? Are we handling it in a way so that you can get less tramp elements?

Part of the tramp elements is unspecific collection. You mix everything. Tramp elements during use (dirt). Shredding, mixed particles. Physically up to a certain level. Incompatible alloys that are put together. Alloy specific sorting is not easy. Copper particle from aluminium stream is easy, but if it is inside another aluminium alloy it is difficult to see it. Physical aspects: density, colour. Sensors that can detect. Two materials that are very close together.

Motor problem is interesting. How much convenience do you need to be happy? Be happy with less stuff – consumer behaviour. Motors in cars is brought in as a convenience. Is it needed? A car is almost a computer – a lot of sensors, wires, cables, electronics. With changing product composition. Think how we recycling and pre-treat them. What is really important if we value our materials? Slow down – life time extension. Narrow the circular economy volume, the volume material that we put into society. System change that will shock the industry.

Shredding and sorting – selective dismantling. Contaminated fractions. Demolish in a more sensible way. Can we take things out before demolishing. WEEE legislation. EU sets the bar. The directive leaves room for the governments to push beyond. The minimum requirements in the EU legislation are not enough and national governments can push a bit harder. The recyclers will need to adapt. Payed by the manufacturers through a negotiation process. Extended Producer Responsibility is happening but very slow. So many actors need to move and that is a challenge. Actors are becoming more active.

Sharing information. EU focuses on data spaces in different sectors. Block chain and traceability – Product Passport. WEEE forum – electronic manufacturers share information that help dismantling and sorting. IP protection culture, confidentiality. What do you need and for what purpose? If there is a specific purpose maybe they are more willing to share data. Give companies control of their data, they know who is looking at it. Batteries passports.

Early adaptors that see the business case. Some others need legal push and stricter policies. Policies are always slower, they are reactive and in a way generic. It takes a long time to get all stakeholders align. NGOs, public outcry, consumer activism in the shorter term to push. Concern with the cobalt and conflict minerals in the battery industry is what started the passport. There is value in selective manual work. The cost structure is not very E-tax (manual labour is very costly). Shift the tax burden from labour to raw materials, so it becomes cheaper to invest in dismantling where you need people. If you use a lot of resources you are going to pay more. Dismantling before shredding is very important to remove some of the dangerous elements.

Everything is very cost focused right now. So the small in-betweens are not implemented. Can you broader the system boundaries to make things profitable? Maybe in the grander scheme is profitable, can be co-financed. Maybe recovering everything is not economic feasible and the criteria are not the same. Policies and regulations can play a role at a certain level.

In aluminium paint is a big problem – carbides. Alloy problem is challenges is similar in all metals. If you mix the wrong alloys it is a very big problem. Design-for-recycle products needs to not be accumulated with the rest of the products cause else you lose the benefits. Separate flows?

Logistics in collection – separate collection flows to do batch processing? 80-20 rule?

White goods. Fridges usually have a different flow where they take our the cooling. There are some separation of materials, different flows for different kinds of products. But mix white goods with cars. Cars and EVs have slightly different flows to remove the battery but then everything goes together. IT goods are different for cars. There is some specialisation going on. Production waste can also get shredded. Everybody tries to do as little as possible. Shredding and sorting is an expensive process. Going to smaller particles doesn’t always make things better. Everything is very case specific. Some companies sell things to other specialised organisations. Energy usage of shredder goes exponentially high with reduction in particle size. The smaller the size the more energy it takes. Smaller is not always better is that sense – find the balance.

Industry looks for stable processes. Dealing with diversity they try to find with a common way of operating for all of them. Constant bandwip necessary in concrete from slag. Specifications make it very difficult. Requirements become narrow and narrow – customer demand is very exigent. Some demands are from standards made in a different time and it is there and it stays. More difficult to meet standards with contaminated scrap. Something in between standard and tailor made sorting. Similar product procedure and adapt. Batch-processing, very operation specific. In circular economy everything becomes a product even when it really is waste. Flows are moving all the time depending on demand. Major trends over time. Scrap and sorting companies are riding these waves. There will always be changes, variations even coming from the same customer. Technological issues to identify some impurities. Some might not be easy to detect and sample.

**SUMMARY ON-SITE VISITS**

*On-site visits were always conducted together with other researchers.*

**Visit to a steel mill scrapyard (observation)**

**Attendees: R&D Expert Metallurgy, Sourcing manager**

The scrapyard is divided of distributed into different spaces where scrap is stored according to internal categories (grades). The separation between these blocks is in most times a cement wall, but that does not imply that neighbour bulk piles are slightly mixed. The grades are not analysed before being put in a specific group, it goes directly from the arrived material from supplier to its space in the scrapyard. The specific amount of a certain grade accepted at the scrapyard depends on the kg/space left for it to be stored. There is a pile also for slag, which is treated by another company.


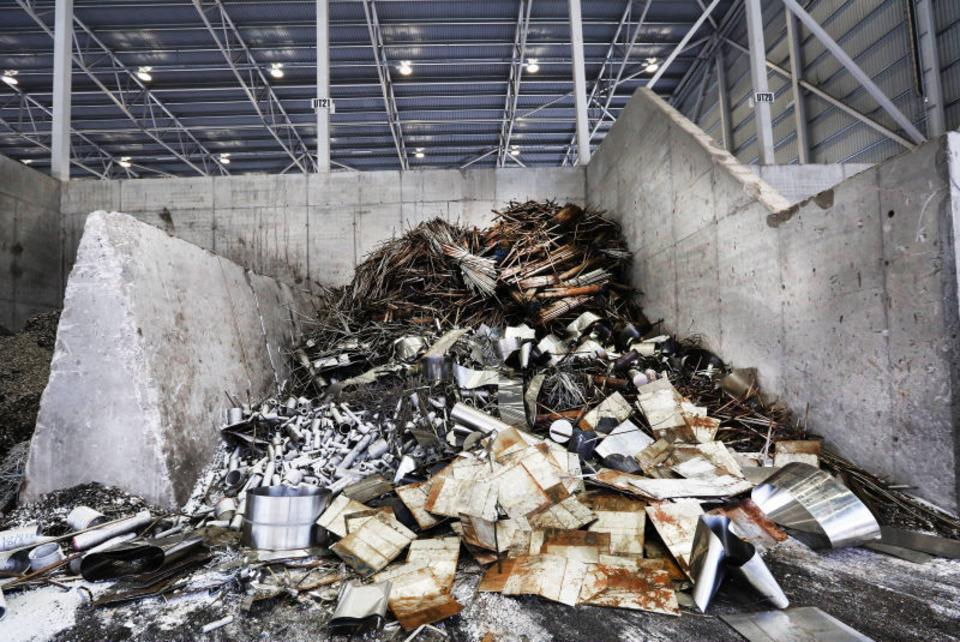


**Visit to a steel mill scrapyard (observation)**

**Attendees:** **Manager of the scrap yard in steel mill**

Material is pressed in cubes for higher density. External scrap comes in lower density and lighter forms, which a lot of air in between. This is not wanted in the furnace. Residual steel from the converter is stored and used in the scrapyard. There is scrap coming from the sampling department (analysis and experiments8 which is a mix of different grades. This is thus used in small amounts as it is very contaminated.

**Visit to a shredder (observation)**

**Attendees:** **Manager of the scrap yard in recycling plant/shredder, Manual dismantlers.**

Radioactive material is checked when the material arrives through sensors. Automobiles tend to have a life expectancy of 16 years. Complex and problematic hybrid cars with electrical material and gas engines that can be very dangerous. Safety is critical, estacially cautious with enw cars. They extract the hazardous material and also what is sellable. However, if it takes a lot of time to extract products they don´t do it. For instance, some wheels or the small motors and cables. It is not worthy to do it. However the quality demands are triggering them to do so. Future plans: (now in an experiment scale) They check te electric motors, cables and plastic. !! Plastic is critical here, there is PP outsime, PC in the lights. ABS and PE in the tank. Monomaterials are wished to be separated as the EU requires higher recycling of plastics. However, complex composite mixes (laminated composites to metals) and additives are difficult to extract. This are mostly inside the car and it is not worthy to extract them because you can´t really recycle them afterwrds. Only monomaterials are recyclable in an economic way. Mixed materials (polymeres) are the problem. The metal in wheals (wheel rims) gets directly sold. They separate the steel ones and the aluminium ones but they do not go to the shredder. It doesn´t make sense to shred them as you would mix rubber, aluminium and steel together, so it is worth to sort it. It can happen that sometimes a wheel gets in if it was not worthy to remove it. Maybe it had a lot of corrosion.

!! High alloyed are a big problem if they get into the shredder. They are non-magnetic so you don´t want them in the steel fraction either way as it has many alloys.

The shredder sells per tonn of material/product. The whole can (product) goes to the shredder, there is no crushing or reduction is size beforehand. If the product seems to be too thick and not be able to go in the shredder, it will be separated and then sent to another material stream. It will be left on the side and sold after.

The rest material from the process is sent to a waste treatment facility. Non-ferrous goes through eddy current separation: Al, Cu, high-alloys and plastics. They all stay in the same pile (as non-ferrous). The non-ferrous are separated in two piles after their thickness. The high-alloys go to the non-ferrous.

Ferrous material also in two piles after thickness. This is directly sold after the shredding process.

Paint can be seen in cans, the big products usually get paint off in the shredding process. Other material (f.e. the material does not enter the shredder – monomaterial) is only crushed and therefore the paint is still there.

!!Meatballs: small electromotor and copper for instance. They need to avoid them from happing with better pre-sorting.

The magnetic rotor sends the magnetic materials up and the non-ferrous down. The rest material (rubber, glass, plastic, wood) gets sold together to another recycler. Two piles separated by size.

Incineration plant waste comes and gets directly to the shredder. This needs to be better sorted in the future. All input material goes together.

!! It is all rules by the demand. MONOMATERIALS. Monomaterial input goes only to presssing machine (in cubes). It mostly comes from industrial plants and it can contain paint. Just pressed NOT shredded. Steel wheels separated from aluminium wheels.

Biggest pressure from green steel in providing quality metallic and non-metallic and on recycling plastic. They work on external pressure.
